# Getting Started in Gene Orthology and Functional Analysis

**DOI:** 10.1371/journal.pcbi.1000703

**Published:** 2010-03-26

**Authors:** Gang Fang, Nitin Bhardwaj, Rebecca Robilotto, Mark B. Gerstein

**Affiliations:** Department of Molecular Biophysics and Biophysics, Medical School, Yale University, New Haven, Connecticut, United States of America; Princeton University, United States of America

## Introduction

The latest innovations and rapid progress in sequencing technologies have substantially enriched whole genome data. Each genome consists of a unique gene inventory, which determines the specific phenotype and interaction with the environment. After 3.5 billion years of evolution, the number of species has expanded considerably [1]. These species originated from simple life forms and have been confronted with complicated environmental changes. These variations, as a result of natural selection, are encoded in their genomes and provide clues to their genetic divergence from a common ancestor. The inference of variations between species by analyzing compositions of gene inventories therefore opens the door to the rich branch of comparative genomics.

One of the fundamental issues in comparative genomics relates to the “causative consequences” of the presence or absence of certain genes in genomes. Before dealing with this issue, we first need to reconstruct evolutionary relationships between genes in different species, and then determine whether given genes have the same function(s). Many complicated evolutionary processes, such as gene speciation, duplication, and horizontal gene transfer make this reconstruction a nontrivial task. Events like whole gene deletion, and gene fusion and fission introduce additional complexity. However, all the evolutionary processes in principle could be uncovered by a phylogenetic tree [2].

Almost all evolutionary events that we identify today through genome comparisons indicate that a specific selection pressure is at work. Selection pressure on certain genes could be so strong and everlasting that the gene could be present in all extant species, or it could be highly transient or specific to certain species, which indicates gene deletions occur widely on phylogenetic trees. This selection pressure on a gene, revealed from its evolutionary history, is determined by the role played by the gene, i.e., its biological function. The known conservation of a gene's sequence coupled with the knowledge of the timing/dating of evolutionary events provides clues about the gene's function. If a gene is preserved in all species with high sequence similarity and there are only a few duplication events along its evolutionary history, we have high confidence that its orthologs have the same function in different species. On the other hand, a large number of duplications and/or deletions along a gene's evolutionary history could indicate neofunctionalization and/or nonorthologous gene displacement [3], and consequently, orthologs in different genomes may have different functions. These facts highlight the significance of function-oriented ortholog identification. In this article, we will review the general procedures to identify orthologs and make ortholog groups. We will focus on the functional analyses of orthologs, review previous work to assess functional consistency of orthologs, and make suggestions to construct better ortholog groups. Lastly, because orthologs can only be identified when the whole gene inventories from all the involved species are examined, the distribution of identified orthologs among species is an immediate result of looking into the composition of ortholog groups. Composition of ortholog groups, which bears important information for downstream research and applications, will also be briefly discussed.

## Ortholog Identification

Orthologs are defined as genes in different species that have evolved through speciation events only. Paralogs, on the other hand arise by duplication events [2]. It is generally assumed that orthologs have the same biological functions in different species [4], and duplication makes room for paralogs to evolve new functions [5]. Identification of orthologs accomplishes two goals: delineating the genealogy of genes to investigate the forces and mechanisms of evolutionary process, and creating groups of genes with the same biological functions. While both are equally important, we focus on the latter in this review: functional analysis of orthologs.

A function-oriented ortholog group consists of orthologs that play the same biological role in different species and also includes recent paralogs with the same biological function, also known as “in-paralogs” [6]. Construction of ortholog groups is fundamental to many objectives, such as transferring annotation to newly sequenced genomes, and pathway comparisons across species [7]. So, not surprisingly, there have been many projects, over the last decade aimed towards creating ortholog groups. According to their construction approaches, these ortholog resources could be classified into two categories: ones that cluster pairs of genes with the same biological functions, and the others that use phylogenetic trees to identify functional divergence events. We briefly discuss both these types in the following sections.

### Ortholog Groups Based on Clustering of Functionally Identical Gene Pairs

**Figure 1:**
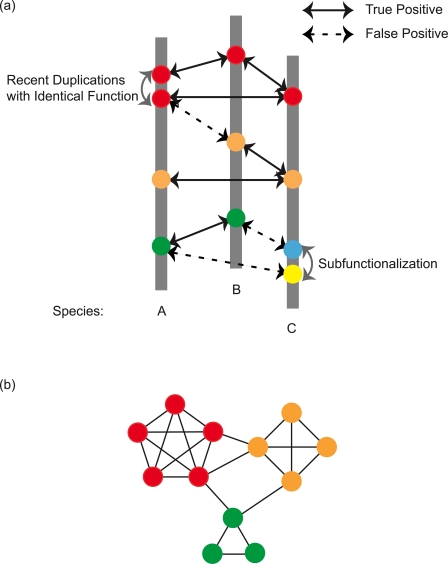
Using BBH strategy to identify functionally identical genes. (a) Three grey vertical bars represent three different species. Circles on each bar represent genes belonging to that species. Colors of the circles indicate a certain biological function; same colors indicate the same biological function. Black bi-directional arrows represent BBHs: a solid BBH arrow means a true positive, i.e., it links two genes with the same function, and a dashed BBH arrow means a false positive, i.e., it links two genes with different functions. Grey curved bi-directional arrows represent gene duplication. Genes are arranged into three tiers on the panel. The top tier is a group of four red circles representing four genes with identical functions. There is a recent gene duplication event in species A, which creates two paralogs (two red circles on the left bar) with the same biological function. In the middle tier, there are three orange circles, which should have been all connected by true positive BBHs. However, if the function corresponding to the orange circle has some relationships with that corresponding to red circle at the top tier, the orange gene from species B and a red gene from species A are detected as a pair of BBH. This is an example of false positive, which is shown as a dashed BBH arrow. The bottom tier includes four genes. The two green genes from species A and B is a pair of true positive BBH. There is a duplication event that caused a subfunctionalization event in species C, i.e., the original green function is shared by the blue and yellow functions in this species. Green gene from species A is connected through a BBH linkage to the yellow gene in species C, but their function are not identical. Similarly, green gene in species B is connected to blue gene in species C. In this tier, subfunctionalization results in two false positive BBH linkages. (b) A network showing the topology of a plausible ortholog group. Nodes are genes and edges are BBH linkages. There are three different functions in this ortholog group (indicated by the three colors). Further partition work is required.

To construct this category of ortholog groups, we first need to identify pairs of genes with the same biological functions, and then cluster them to make functionally consistent ortholog groups. Such gene pairs are usually detected by using the Bi-directional Best Hit (BBH) strategy. As the name suggests, a pair of BBH genes are two genes that are reciprocally most similar to each other when considering all the genes from that organism [4]. The basic assumption behind regarding a BBH pair as a functionally identical gene pair is the following: If a certain function is required in two different species (e.g., attaching alanine to its compatible cognate tRNA by alanyl-tRNA synthetase), it is most likely that this function is carried out by a pair of the most mutually similar genes from these two species. This assumption is true for many cases, i.e., a BBH links two genes with the same biological function together (Figure 1a). There are, of course, cases that breach this assumption. This assumption includes two essential elements: “function by single gene” and “present in both species.” Various evolutionary events can conflict with the two elements. For example, the concept of “function of a single gene” can be destroyed by a subfunctionalization event [8], i.e., a gene's function in one organism is realized by two genes (which could be a pair of paralogs) in another organism. In this case, a plausible BBH links the two genes with related but not identical functions (false positives, Figure 1a). The latter element, “present in both species,” will be violated in case the function is not required in one species, or there is a compensatory pathway. There could be other more complicated evolutionary events for a BBH linkage to exist between two genes with different functions, hence increasing the false positive rate. False positive BBH linkages cluster genes with different functions into the same ortholog group, thus breaching the functional consistency of ortholog groups. On the other hand, if there is a pair of recently duplicated paralogs that have not acquired new functions yet, then by using BBH-based approaches, we will miss at least one gene in the ortholog group (false negatives, Figure 1a). So, we need to be careful while identifying genuine BBH pairs that connect two genes with the same function and clustering genes into ortholog groups by BBH linkages.

The probability of missing a gene in an ortholog group can be kept low by including a sufficient number of species. At the same time, increasing the number of species, especially phylogenetically distant species, could introduce more subfunctionalization and/or neofunctionalization (genes evolving new functions) events, thus increasing false positive rates by including many BBH pairs that are not functionally identical genes. In the clustering step, such false positive BBHs could result in functionally different ortholog groups being connected together in a network (Figure 1b) where genes are depicted as vertices and BBH linkages as edges. This network is referred to as the issue of transitivity of BBHs in ortholog group construction [9]. Transitivity, a property of orthologs, implies that if genes A and B are orthologs, as are genes B and C, then A and C should be orthologs as well [9]. However, constructing ortholog groups simply by joining BBHs together tends to include genes with different functions. Therefore, the transitivity issue is a major challenge in accurately constructing BBH-based ortholog groups. To deal with the transitivity issue, we can set thresholds for the similarity of two genes in the first step of detecting BBH, to reduce the false positive rate. This threshold can be any combination of the similarity score, alignment E-value, and/or difference in gene lengths [10],[11].

Evolutionary and biological knowledge could also contribute to the construction of ortholog groups. For example, Inparanoid [6] introduces an evolutionary outgroup species to evaluate a BBH in the following way. Given genes A and B from two species that form a pair of BBH, if another gene C from an outgroup species is a BBH to both A and B, then BBH linkage of A-B should be stronger than those between A-C and B-C. If not, the linkage of A-B is likely to be a false positive [6]. As another example, eggNOG [12] detects events like gene fusion and protein domain shuffling that might lead to functionally distinct ortholog groups to be linked together by comparing protein domain architectures using databases like Pfam [13] and SMART [14]. Similarly, in the clustering step, there have been several attempts to purify ortholog groups. For example, a simple but seminal idea to tackle the transitivity issue is to use complex linkages instead of a single BBH, as used by the COG method [4], where a set of three genes, with each pair forming a BBH makes up a minimum COG and two COGs are joined together if they share a common BBH. Following this method, when a gene joins an ortholog group, not only must it have two genes in the group as its BBH, but also the two genes themselves must be BBHs of each other. The COG method indicated that single linkage BBH clustering is not as reliable to build functional consistent ortholog groups and pioneered the idea to build BBH-based ortholog groups using a clustering method. However, while the COG method works quite well for most bacterial genes, it is not very applicable to eukaryotic organisms [15]. This difference is probably due to the much higher gene duplication rates, and hence higher subfunctionalization/neofunctionalization in eukaryotic organisms [16]. To address this issue of frequent functional divergence, if a three-way BBH linkage is not enough, more densely connected BBH linkages can be created. OrthoMCL is a good example that implements this clustering strategy [17]. Following this idea, genes are clustered, and their distances are measured by the BBH linkages. The distance amongst a pair of genes could be 1 or 0, depending upon if a BBH exists between them or not, respectively. We can also quantify this linkage to differentiate between strong or weak BBH linkages by using the sequence similarity score between the two genes. OrthoMCL used the *p*-value of protein alignments as the distance [17]. Note that when we quantify BBH, we might introduce some biases that need to be normalized. For example, amongst genes that underwent recent duplications in a genome, the sequence similarities or *p*-values of their alignments could be very significant, although these quantities might not genuinely reflect a strong selective pressure as compared to two orthologs that speciated a long time ago and have high sequence conservation [17]. Once the biased gene distances from the same genome are normalized appropriately, several clustering algorithms can be used, for example hierarchical clustering, to group genes into ortholog groups, although it has been suggested that some method like the Markov Cluster Algorithm is more efficient [17].

Besides these works, there are some other ortholog group resources worth discussion, such as OMA (Orthologous MAtrix project) [18] and Roundup [19]. OMA covers 352 species ranging from bacteria to eukaryotic organisms. In addition, it emphasizes the importance of using global sequence alignment in BBH identification, which reduces the possibility of a false positive BBH owing to sharing common protein domains [18]. Roundup uses an upgraded method of BBH, Reciprocal Smallest Distance (RSD) [20], to identify the functionally identical gene pairs among species. Similar to BBH, RSD also picks a pair of genes that are mutually most similar one to each other, but instead of using sequence similarity, RSD uses evolutionary distance (estimated number of amino acid substitution) to measure similarity between proteins, i.e., a pair of genes with the smallest reciprocal distance is identified with the same biological function [20]. Beyond this, Roundup provides user-friendly data presentations at their website, which facilitates functional and phylogenetic analyses of ortholog groups [19]. We list websites of the above mentioned ortholog resources, with several others, in Table S1. Each one has their own specific strategies to handle BBH linkages or clustering. Note that in many of these works, BBH not only refers to a pair of genes from two different species, it can also refer to a pair of mutually most similar genes from the same species. This strategy is to assure recent duplications are considered in formation of ortholog groups. Finally, BBH-based methods are all quite efficient in terms of computing resources.

### Phylogeny-Based Ortholog Groups

Another category of ortholog groups is based on phylogenetic trees. Phylogeny is the evolutionary history of species, and it is usually shown as a phylogenetic tree that also describes the evolutionary relationships between species. Phylogenetic trees are also widely used to show how a gene evolves. Being computationally expensive, phylogeny-based methods were not applied to large-scale ortholog group construction earlier. Recently, some automated unsupervised phylogenetic tree construction algorithms have been proposed, leading to several phylogeny-based ortholog resources, such as PhylomeDB [21], PANTHER [22], TreeFam [23], and Ensembl Compara [24].

**Figure 2:**
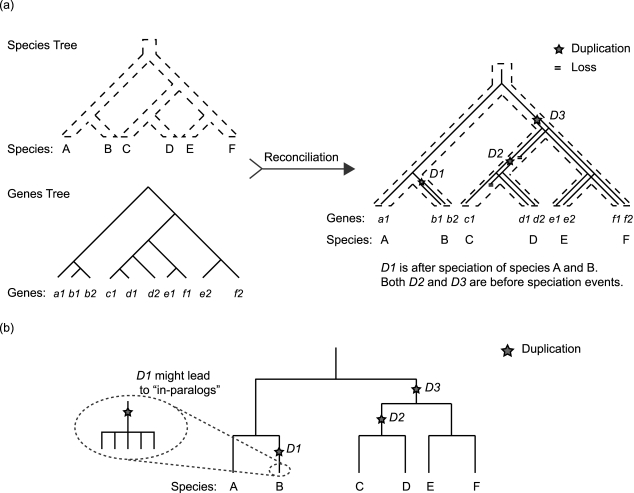
Phylogeny-based ortholog group construction. (a) On the upper left panel, a tree delineates the phylogenetic relationships among six species, A–F. Below the species tree, a phylogenetic tree is shown, which includes ten genes taken from the six species. The right panel shows the tree after reconciliation, which is the process of comparing the gene tree with the species tree to date evolutionary events like duplication and deletion. For the reconciled tree, the dashed thick lines represent the species tree as the same as the one on the upper left panel, and solid lines indicate the reconciled gene tree. Three duplication events are dated. Duplication D1 occurs after the speciation of species A and B. D2 occurs before speciation of C and D, and D3 occurs before CD and EF. According to current tree analysis algorithms, functional partition points will be at D2 and D3. (b) Gene duplication close to leaf nodes does not necessarily result in function divergence. The schematic shows the evolutionary history of the same gene, with the only difference that the tree includes five closely related species of B, instead of one, where duplication D1 occurs before speciation of the five B species. D1 is so recent that it is hard to estimate if there will be subfunctionalization/neofunctionalization. It might result in “in-paralogs” where duplicated genes in all five B species have the same function. D2 and D3 are duplications that happened a long time ago. If paralogs due to D2 and D3 are present in most descendant species, there is a higher chance for them to have diverged biological functions.

The approach behind building phylogeny-based ortholog groups is straightforward: analyze the topology of a phylogenetic tree to identify a branch of genes with consistent biological functions. The basic idea is to build phylogenetic trees for candidate genes, followed by reconciliation of gene trees according to the species tree in order to date duplication and deletion events on the gene's evolutionary history (Figure 2a). On the basis of such events, we can estimate when a gene's function had diverged on the tree and can identify a branch that could be regarded as a functionally consistent ortholog group. There is a large amount of literature discussing the analysis of phylogenetic trees (their reconstruction and reconciliation) in addition to some software and tools available for tree reconciliation, such as RAP [25], SYNERGY [26], and TreeBeST [23]. Obtaining the correct gene phylogenetic tree and performing a suitable reconciliation is crucial for ortholog group construction. A detailed discussion of these steps is beyond the scope of this review. Our focus here is to discuss building functionally consistent ortholog groups for large-scale genome data analyses. In this regard, the selection of genes to build the tree, identification of internal nodes (nodes other than leaf nodes) indicating function divergence, and partitioning the tree at such internal nodes turn out to be major and important tasks, which are discussed in the following sections.

In spite of tremendous advances in computing technology, it is still not very easy to construct phylogenetic trees with thousands of genes. On the other hand, the purpose of building a tree here is to determine gene duplication events that result in function divergence. It might not be useful to build trees on the basis of extremely highly conserved genes so that such events could not be detected on the tree. Therefore, we need to carefully choose the scale of divergence of genes to be included in the tree. For some phylogeny-based ortholog groups [21],[23], genes clustered by single BBH linkage are selected as candidates to make a phylogenetic tree. From this point of view, partitioning phylogenetic trees also becomes a step in making BBH clusters. For some specific BBH clusters, such as bacterial transcription regulators, the number of genes included is beyond the current capacity (over 30,000 coding sequences from about 600 bacterial genomes are clustered into one single group by BBH linkage, unpublished data), and appropriate preclustering processes have to be carried out. Once the tree is built, determining the location of the functional divergence cut is a rather subjective decision. If duplication is closer to the root of the tree and the tree covers species with enough divergence, we have a higher confidence in splitting the two duplicated lineages and making two different ortholog groups; we can do so because it is less likely for a large number of species to retain two paralogs with the same function. On the other hand, if duplication is closer to the leaves, then on the basis of the tree topology, we cannot determine if this duplication event will lead to a subfunctionalization/neofunctionalization event with high confidence.

There have been several attempts to perform automated function-oriented partitioning of a phylogenetic tree. Though their underlying algorithms vary, the basic premise remains the same: duplications that occurred before any speciation are all regarded as events leading to functional divergence (Figure 2a) [21],[26]. This strategy, however, has some drawbacks. It makes ortholog groups depend on how closely related the species are, and the partition strategy is somewhat stringent, as duplication that occurred before internal nodes close to the tree's leaves does not necessarily indicate functional divergence (see Figure 2b). However, currently, this is the best method to partition a phylogenetic tree due to several reasons. First, reconciliation of gene trees is often so erroneous that many duplication events at internal nodes are not unequivocal. This finding is especially true for multicellular eukaryotic organisms in which many gene duplications are conserved [27]. Second, there is no universal time reference for all the ortholog groups to decide if a duplication event is old enough to partition the tree. Because the selection pressure for different genes is different, a good way to estimate functional conservation based on the topology has yet to be found. Obviously, these challenging issues associated with phylogeny-based ortholog group construction have already been noted and efforts have been made by the research community to address them. For example, to improve the quality of the data, TreeFam, manually curates some ortholog groups on the basis of literature and the examination of each tree's topology [23]. As another example, PANTHER [22] manually identifies functionally divergent internal nodes of a gene family tree using not only phylogenetic relationships (e.g., duplication events followed by relatively fast sequence divergence), but also curated functional information about each gene such as Gene Ontology (GO) annotations [28] and descriptions from SwissProt [29].

In spite of the aforementioned issues, we believe that a gene's evolutionary history is essential to study evolutionary mechanisms and to understand the selective pressure and function conservation and/or divergence of the gene. However, generating automatic biological function interpretations from a gene's phylogenetic tree is just starting to be addressed. The real events that occur during a gene's evolutionary history could be much more complicated than just a combination of duplications and deletions. Due to space limitations, issues about the accuracy of a phylogenetic tree itself are not discussed here. We list some phylogeny-based ortholog resources in Table S1.

## Functional Assessment of Ortholog Groups

### Ortholog Group Benchmarking Using Functional Genomics Data

As many ortholog group resources are constructed, it is necessary to assess their accuracy. The assessment of biological function is not a simple task, because the accurate function of protein can only be unambiguously explored by biochemical and/or structural studies. It has been impossible to perform independent experiments for all genes one by one, species by species. However, there is a wealth of genomics data, which can be used to benchmark ortholog groups. For example, large-scale gene expression data from different species: if expression profiles are significantly different among orthologs in different species, it would be less evidential that identified orthologs have the same biological function. There is a recent analysis that systematically harnesses functional genomics data to examine the accuracy of ortholog predictions [30]. In that work, Altenhoff and Dessimoz made comparisons between OMA and several other resources including the above-mentioned OrthoMCL, Inparanoid, and Ensembl Compara, etc., using GO terms, enzyme (EC) number category, gene expression profiles, and gene neighborhood conservation. In their calculation of GO terms' consistency among orthologs, a pair of orthologs is assigned a score ranging from 0 (unrelated) to 1 (identical GO term) according to the hierarchical structure of GO terms and their frequencies [30],[31]. The average values of such scores are calculated for different ortholog resources. A higher score indicates a more functionally consistent ortholog group. GO term comparisons show that when focusing on function identity (specificity), simple BBH-based ortholog resources outperform the others. However, as the authors point out, GO itself is largely constructed on protein sequence alignments. It should be noted that this calculation might lead to biases because of circular dependency [30]. Similar comparisons of EC number consistency are performed across different resources, and Inparanoid outperforms others. It is rather surprising that none of the ortholog resources shows significant correlation between orthologs and gene expression profiles, using human and mouse gene expression data [30]. The observation that orthologs have different gene expressions in human and mouse is probably due to the sophisticated regulatory difference between the two species, and/or it could also mean there is more room to improve the construction strategies of functionally consistent ortholog groups. Lastly, conserved synteny is explored to see if there is correlation with orthologs. It has been shown that adjacent genes are more likely to have related biological functions [10], so it is assumed that if two genes are orthologs, their neighboring genes from different species are more likely to be orthologs. Comparison of conserved synteny also supports that simple BBH-based algorithms provide more functional consistent orthologs [30]. In this work, authors analyzed phylogeny of orthologs as well, which shows OMA most accurately presents the evolutionary relationships between genes, even though OMA is not based on phylogeny [30]. Additionally, several other works also examined whether GO terms and EC numbers from different species are consistent within the same ortholog group, and evaluated the accuracy of orthologs in terms of conserved synteny and evolutionary history [32],[33]. Beyond the functional genomics data, protein–protein interaction data could also be integrated in this assessment work [33]. These comparisons and estimations of quality using functional genomics data highlight the individual advantages of each ortholog resource. As we focus on functional consistency among orthologs, the BBH-based ortholog resources producing high specificity are suggested for the downstream analyses.

### Incongruence between Ortholog Resources and Suggestions for Possible Improvements

**Figure 3:**
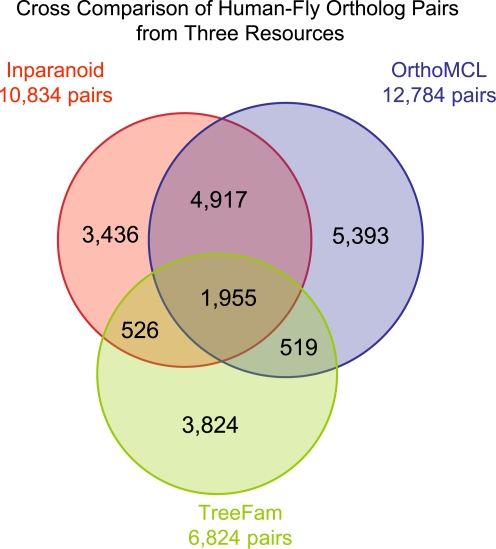
Cross comparison of human-fly ortholog pairs from three different ortholog resources: Inparanoid, OrthoMCL, and TreeFam. Due to the asynchronous updates of these data resources, the gene sets used in the three are slightly different. To make a cross comparison, we mapped their gene IDs to the most recent human and fly gene IDs in Ensembl 53, using biomart (http://www.ensembl.org/biomart). After ID mapping, we got 10,834 pairs of human-fly ortholog genes from Inparanoid, 12,784 pairs from OrthoMCL, and 6,824 from TreeFam. Intersections of the three pairs sets are shown in the Venn diagram. Among these ortholog pairs, only 1,955 pairs of orthologs exist in all three ortholog resources, accounting for 18% of Inparanoid human-fly ortholog pairs (15% and 28% in OrthoMCL and TreeFam, respectively). Details of this and other orthologs comparisons can be found at http://wiki.gersteinlab.org/pubinfo/Ortholog_Resources.

Not only are there large inconsistencies when mapping different ortholog resources to the same functional genomics data, the cross comparisons of different ortholog resources themselves also show significant differences [24],[30],[34]. If we define congruence of ortholog groups as a state of containing exactly the same gene sets, many of the above-mentioned resources have less than 50% congruent ortholog groups between them, and when more remotely related species are considered, the overlap is even lower (for example, see Figure 3). Why are there such differences? This question requires careful study, as deeper understanding of the error-prone steps in various algorithms could trigger developments toward better ortholog groups. For the BBH-based algorithms, as we discussed in the first section, the major challenge is how to reduce false positive BBH linkages. We can focus on the inconsistent sets of orthologs between different ortholog resources and start the analysis by asking some basic questions. How many BBH pairs from the two species are not functionally identical? Does the number of BBHs with different functions vary between closely and remotely related species? If they do, is there some correlation between phylogenetic distance and the number of false positive BBHs? If such a correlation exists, could it be used in the clustering of BBH pairs? Do such pairs have any GO term preferences? What are the genes that are always ambiguous in ortholog group construction? Can we introduce more stringent or relaxed criteria for certain ortholog groups? Such questions are helpful in providing clues about how a gene's function evolves.

For the other category of ortholog resources (phylogeny-based), the underlying idea is that duplication leads to subfunctionalization/neofunctionalization such that two paralogs play different roles. We can assume it to be true for most cases, but there are exceptional cases as well. These exceptions provide us good resources to develop some better-founded theories. Since phylogenetic trees delineate a gene's evolutionary history and record all the evolutionary events, there is room for improvement as compared to that for BBH-based approaches. Technically speaking, challenges for BBH approaches center around how to reduce false positive BBH linkages and cluster functionally consistent genes into groups. In contrast, phylogeny-based approaches have many more aspects to consider: (1) selection of genes to build the tree, (2) the accuracy of the tree reconciliation with known phylogeny, and (3) identification of functionally divergent internal nodes. Besides improving the accuracy of the tree, a way to identify a more appropriate function-oriented partition strategy, which is currently somewhat stringent and may separate nodes that are not functionally divergent, needs to be developed. The function-oriented partition issue is highlighted particularly for Ensembl Compara whose specificity is not significantly improved in spite of reporting fewer orthologs [30]. For developing better function partitioning strategies, a few questions need to be answered. Can we take the number of duplication events into account when deciding where to partition the tree? Whether the branch lengths and the similarity of the two paralogs are worth exploring towards identifying recent subfunctionalization/neofunctionalization events? Can we map functional genomics data on the tree and find more clues to locate the partition point? What kinds of tree topologies make the tree difficult to partition? Should we treat the topologically different trees separately? All such questions are just starting to be addressed.

### A Short Discussion of the Definition of Ortholog's Biological Function

A final issue worth discussing is how to define a gene's function [30]. If we have different views about a gene's function, we cannot easily reach an agreement about the quality of ortholog groups. One of the many views is that a gene's function is its relationship with other biological objects in the cell [35], including its interactions with other genes, proteins, chromosome intergenic regions, etc. If we define the gene's function in this way, then a predefined term or several words might not be enough. For example, the gene *dnaE* codes DNA polymerase III α subunit in both gamma-proteobacteria and firmicutes. However, in gamma-proteobacteria, *DnaE* is responsible for the synthesis of both leading and lagging strands, whereas in firmicutes, this subunit only synthesizes lagging strand. Due to this difference, there are >78% genes in firmicutes genomes coding on the leading strand, compared to ∼56% genes in gamma-proteobacteria genomes [36]. It might be more appropriate to assign a list of physical interactions with other biological objects to the definition of a gene's function. The definition of biological function is bound to be controversial, but a discussion in this regard is highly valuable. With the current data, some studies have already been done to explore gene function by conducting large-scale surveys of the conservation of protein-protein interactions (interlogs) and protein-DNA interactions (regulogs) [37]. However, the fallacies of these interaction datasets are well known, such as inconsistencies of protein–protein interactions reported by different experimental methods, and/or across different species. But such issues inspire us to integrate the two seemingly disparate projects: identification of orthologs and the functional genomics of interactions. We can design functional genomics experiments to check the functional consistencies of putative orthologs for species that are evenly distributed on the phylogenetic tree. This way, we can try to set up “gold standards” for orthologs from such experiments [7]. Even if we observe some interaction differences between our putative orthologs, we obtain clues from these differences to understand why some predictions are correct while others are not.

## Composition of Ortholog Groups and Distribution of Orthologs among Species

Ortholog groups contain genes from different species and composition of an ortholog group provides a direct and very valuable factor for downstream analyses: the distribution of orthologs across species. First of all, composition of an ortholog group could give us information about its biological function. For example, if we are going to select “high quality” ortholog groups across species (with a high confidence that the genes in a group have consistent function), from the phylogeny-based ortholog groups, we can select the groups with genes widely distributed on the tree with few duplication and deletion events. Similarly, for BBH-based ortholog groups, we can pick up clusters covering enough species with a dense (close to a clique) BBH-network as the high quality groups.

Genes from such ortholog groups are called persistent genes [26],[38], as they have strong selective pressure, high functional consistence, and indispensability in extant species. An example of the systematic comparison of persistent genes between gamma-proteobacteria and firmicutes set up clear cause-effect relationships between several genotypes and phenotypes, and provide functional predictions and clues for further experiments [38].

A good amount of knowledge can be gained from ortholog groups by comparing their component genes' distribution among species and evolutionary profiles [39]. There are several tools available for such comparisons and one good example is Roundup [19]. Using Roundup, one can explore the co-presence and/or co-absence of genes in a certain clade, i.e., correlation or anticorrelation between genes' evolutionary profiles. There are various biological questions that could be raised regarding genes' evolutionary profiles. Are there unique features associated with clade-specific genes that are preserved only in a branch of species on a phylogenetic tree? On the other hand, could niche-specific genes, which are present in species from a particular environment and absent under other circumstances provide clues about their interactions with environmental factors? Additionally, it is known that some essential biological processes in all organisms are associated with genes only present in some clades. How can we identify candidates in other clades performing the same function? We can use correlation and anticorrelation between evolutionary profiles to narrow down the number of candidate genes or even boost the prediction of genes as experimental targets.

## Conclusion

In summary, accurate ortholog group construction is fundamental to comparative genomics and it accomplishes something beyond the mere purpose of providing high quality data resources for other applications. It deepens our understanding of biology because studying BBH linkage or phylogenetic trees for gene orthology will lead to the combined results of various selective evolutionary events. In turn, selective pressure, explored by sequence or protein structure similarity, is borne by a gene's function. Hence, the study of refining ortholog groups is virtually the study of how genes' functions evolve, remain conserved, and/or further diverge. Many rewarding projects that revolve around this study are waiting to get started on.

## Supporting Information

Table S1 Selection of Ortholog resources.
(0.03 MB XLS)
